# Expectation of clinical decision support systems: a survey study among nephrologist end-users

**DOI:** 10.1186/s12911-023-02317-x

**Published:** 2023-10-26

**Authors:** Fruzsina Kotsis, Helena Bächle, Michael Altenbuchinger, Jürgen Dönitz, Yacoub Abelard Njipouombe Nsangou, Heike Meiselbach, Robin Kosch, Sabine Salloch, Tanja Bratan, Helena U. Zacharias, Ulla T. Schultheiss

**Affiliations:** 1 Institute of Genetic Epidemiology, Faculty of Medicine and Medical Center - University of Freiburg, Freiburg, Germany; 2 Department of Medicine IV – Nephrology and Primary Care, Faculty of Medicine and Medical Center - University of Freiburg, Freiburg, Germany; 3 Present Address: Department of Medical Bioinformatics, University Medical Center Göttingen, Göttingen, Germany; 4 Institute of Computational Biology, Helmholtz Zentrum München, Munich, Germany; 5 Department of Nephrology and Hypertension, University Hospital Erlangen, Friedrich-Alexander-Universität Erlangen-Nürnberg, Erlangen, Germany; 6 Institute for Ethics, History and Philosophy of Medicine, Hannover Medical School, Hanover, Germany; 7 Competence Center Emerging Technologies, Fraunhofer Institute for Systems and Innovation Research ISI, Karlsruhe, Germany; 8 Peter L. Reichertz Institute for Medical Informatics of TU Braunschweig and Hannover Medical School, Hanover, Germany

**Keywords:** Survey, CDSS, Clinical Decision Support Systems, Nephrology

## Abstract

**Background:**

Chronic kidney disease (CKD), a major public health problem with differing disease etiologies, leads to complications, comorbidities, polypharmacy, and mortality. Monitoring disease progression and personalized treatment efforts are crucial for long-term patient outcomes. Physicians need to integrate different data levels, e.g., clinical parameters, biomarkers, and drug information, with medical knowledge. Clinical decision support systems (CDSS) can tackle these issues and improve patient management. Knowledge about the awareness and implementation of CDSS in Germany within the field of nephrology is scarce.

**Purpose:**

Nephrologists’ attitude towards any CDSS and potential CDSS features of interest, like adverse event prediction algorithms, is important for a successful implementation. This survey investigates nephrologists’ experiences with and expectations towards a useful CDSS for daily medical routine in the outpatient setting.

**Methods:**

The 38-item questionnaire survey was conducted either by telephone or as a do-it-yourself online interview amongst nephrologists across all of Germany. Answers were collected and analysed using the Electronic Data Capture System REDCap, as well as Stata SE 15.1, and Excel. The survey consisted of four modules: experiences with CDSS (M1), expectations towards a helpful CDSS (M2), evaluation of adverse event prediction algorithms (M3), and ethical aspects of CDSS (M4). Descriptive statistical analyses of all questions were conducted.

**Results:**

The study population comprised 54 physicians, with a response rate of about 80–100% per question. Most participants were aged between 51–60 years (45.1%), 64% were male, and most participants had been working in nephrology out-patient clinics for a median of 10.5 years. Overall, CDSS use was poor (81.2%), often due to lack of knowledge about existing CDSS. Most participants (79%) believed CDSS to be helpful in the management of CKD patients with a high willingness to try out a CDSS. Of all adverse event prediction algorithms, prediction of CKD progression (97.8%) and in-silico simulations of disease progression when changing, e. g., lifestyle or medication (97.7%) were rated most important. The spectrum of answers on ethical aspects of CDSS was diverse.

**Conclusion:**

This survey provides insights into experience with and expectations of out-patient nephrologists on CDSS. Despite the current lack of knowledge on CDSS, the willingness to integrate CDSS into daily patient care, and the need for adverse event prediction algorithms was high.

**Supplementary Information:**

The online version contains supplementary material available at 10.1186/s12911-023-02317-x.

## Background

Chronic kidney disease (CKD) is a major public health problem with a prevalence of about 10% in high-income countries [1–3]. The prevalence of CKD is rising, especially in older patients [2, 4] and will continue to increase in an ageing society, leading to a higher proportion of multi-morbid patients and higher complexity in patient treatment.

CKD constitutes a complex disease due to differing underlying disease etiologies in each patient that can in turn lead to many complications [3], comorbidities [1, 5], and polypharmacy [6, 7]. CKD is associated with a worse prognosis of adverse long-term patient outcomes due to reduced kidney function itself and due to comorbidities, particularly cardiovascular diseases (CVD) [1, 8], as well as major risk factors associated with CKD progression and increased mortality [9, 10]. A clear overview and differentiated risk calculation of an individual patient’s diseases and possible complications are necessary to avoid adverse events, such as CVD or acute kidney injury (AKI). Optimized treatment requires the consideration of all data levels, from clinical parameters, biomarkers, lifestyle factors and disease history to medication use. Mathematical models can assist in integrating all mentioned data levels. These models recognize underlying data dependencies and their associations with disease progression or risk for complications. Many clinical decision support systems (CDSS) integrate mathematical models based on machine-learning methods to predict different adverse events and can thereby improve management of CKD patients [11–14]. Here, supervised machine-learning algorithms use training data, i.e., pairs of predictor and known outcome variables, to deduce mathematical models which can then be employed to predict an unknown outcome for new data.

Several prediction algorithms in nephrology already exist. The most prominent algorithms have focused on prediction of CKD progression to kidney failure. One of them has been developed in a German CKD cohort (Z6 risk equation [15]) including 4915 CKD stage 1–5 patients with a mean observation time of 3.71 years. The Z6 has been successfully validated in three independent cohorts, including a total of 3063 patients. The other one has been developed within a Canadian cohort of 3004 CKD patients of whom 344 reached kidney failure (KF) during a follow-up of three years and validated within the CKD prognosis consortium (kidney failure risk equation [16, 17]). Here, the developed equation was validated in 31 cohorts from 30 countries in 721,355 CKD patients with 23,829 KF cases during 4 years of follow-up with high discrimination and adequate calibration of the equation concerning development of KF. The former selected six, whereas the latter selected four clinical routine patient parameters as predictors in order to predict the outcome “kidney failure requiring kidney replacement therapy” for CKD patients. Two very specific prediction algorithms can be used in special settings only: 1) A prediction algorithm for IgA nephropathy that can be used in multiple ethnic groups at the time of kidney biopsy [18] developed and validated in 3927 patients with biopsy-proven IgA nephropathy from Europe, North America, China, and Japan over seven years of follow-up; 2) a prediction algorithm for AKI [19], which is based on a retrospective analysis of data from a large number of adult intensive care patients of the EPaNIC clinical trial [20] using 4640 patients to build and validate the predictive models. So far, these algorithms are individual efforts and no overarching framework has been developed making the use of these CDSS somewhat tedious for nephrologists with restricted time on their hands that is bound by acute patient care.

One of two initiatives trying to remedy this issue is a junior consortium in the field of systems medicine (CKDNapp [21]), the other one an effort from the Hannover region (NEPHRO-DIGITAL; [22]), Germany. While CKDNapp (CKD Nephrologists’ app) is aiming at predicting adverse events and disease progression, as well as delivering comprehensive literature support as an easy-to-use software for cell phones, tablets, and desktop computers, NEPHRO-DIGITAL will be an eHealth platform for data sharing between outpatient nephrology, primary care, pediatricians and nephrology clinics and will also incorporate an interoperable CDSS.

Taking these efforts into account, it is of prime interest to assess nephrologists’ attitudes towards a CDSS in order to successfully implement CDSS frameworks into daily medical routine. We therefore aimed to investigate the following user aspects via a uniquely designed survey for nephrologists: 1) experiences with CDSS (module M1), and 2) expectations of a CDSS in general (M2), 3) evaluation of potential features of interest, like adverse event prediction algorithms within a CDSS in particular (M3), as well as 4) opinions on ethical aspects of CDSS (M4).

## Methods

### Survey sample population

The study was approved by the ethics committee of the University of Freiburg and registered at the German registry for clinical studies (DRKS00025054) and reporting was standardized using the modified STROBE Statement whenever possible [23]. Nephrologists were recruited via three different ways: (i) the German Chronic Kidney Disease (GCKD) study coordinating center by using contact information of collaborating nephrologists, (ii) a public presentation at the German society of nephrology conference, (iii) published articles in two magazines for nephrologists involved in CKD out-patient care [24, 25]. Nephrologists collaborating with the GCKD study were contacted via telephone by study personal and interviewed directly, if they were willing to do so. Conference attendees of the German society of nephrology conference were able to directly take the survey by a provided QR code on one of the slides and were also encouraged to do so. Nephrologists reading the provided articles on the in two magazines were able to take the survey via a provided link or QR code at their leisure or could also contact the study center for a telephone interview. Nephrologists from all regions across Germany were thereby recruited for the survey, since the GCKD study is a study working with nephrologist all over Germany, the conference of the German Society of Nephrology adheres to nephrologists in all of Germany and both magazines are available to any nephrologist in Germany. Any nephrologist with experience in CKD out-patient management was therefore able to participate after giving informed consent. There were no decided exclusion criteria. All participants gave informed consent prior to taking the survey. All methods were carried out in accordance with relevant guidelines and regulations following the Declaration of Helsinki.

### Survey questionnaire

The survey was conducted either by telephone or as a do-it-yourself online interview from 08/2021 to 03/2022. The telephone interview was conducted by trained interviewers. The survey was developed as a 38-item questionnaire with a processing time of approximately ten minutes and included a short introduction giving a definition of CDSS in general and prediction algorithms in particular (Additional file 1: Supplemental Table 1). First, information on socio-demographics, e.g., age, gender, years of ambulatory work experience, or number of CKD patients treated per day on average, was collected. Subsequently, questions from four modules were asked: (M1) the physician’s experience with CDSS in general; (M2) nephrologists’ expectations toward a helpful CDSS; (M3) evaluation of the importance/usefulness of prediction algorithms and literature support within a CDSS; (M4) ethical aspects with regard to CDSS. The very last question asked for the willingness of nephrologists to participate in a testing phase of a uniquely designed CDSS, once a first version of the application has been developed.

The survey was available in German language only since the survey was conducted in Germany. (Additional file 1: Supplemental Table 1). All questions were included based on suitability after a literature search on the topic of CDSS. Here, previous studies have identified the “perceived usefulness” and “perceived ease-of-use” to be particularly important for the acceptance or declination of innovations [26, 27] as part of the frequently used extended Technology Acceptance Model (TAM2) [28–30]. Additionally, the physician’s willingness to change his/her workflow and the future compatibility of the CDSS to daily work practice were included in the questionnaire [26, 28, 31]. Response formats included the Likert scale (five-point: 1: does not apply at all; 2: does rather not apply; 3: partly applies; 4: largely applies; 5: fully applies; or 1: unimportant – 5: very important, respectively) or multiple choice with preselected options for closed questions.

### Data collection with REDCap and data privacy

Development of the questionnaire, study data collection and data management were carried out using the Electronic Data Capture System (REDCap) [32, 33]. REDCap is a well-established system for translational research and has been described before. Participants’ answers were collected anonymously. In order to preserve data protection, no location of activity was requested. Furthermore, the collection of socioeconomic data was categorized to prohibit recognizability of participants.

### Statistical analysis

Descriptive analyses of all queried items were conducted. Visualizations of answer profiles per question included histograms, pie charts, bubble plots, and radar charts accessible at (www.ckdn.app/survey). Software tools included, on the one hand, pre-established workflows within REDCap, giving out basic barplots, scatterplots, pie charts, and basic statistics on missingness of variables, on the other hand, Stata SE 15.1 (StataCorp. 2017. *Stata Statistical Software: Release 15*. College Station, TX: StataCorp LLC). A radar chart, bubble plot as well as modified pie chart were created for Modules 1, 2, and 3 using the software Excel (*Microsoft Office, Release 16*).

## Results

### Demographics

Our study population comprised 54 nephrologists from all across Germany. All survey questions were fully completed by 30 nephrologists (56.6%). Missing responses ranged from *N* = 0 to *N* = 11, resulting in a response rate of about 80–100% per question (Additional file 2: Supplemental Fig. 1). Participants were mostly aged between 51–60 years (45.1%), followed by the age group of 40–50 year olds (27.5%). Age groups of participants being older than 60 years or younger than 40 years were almost equally present (13.7% and 11.8% respectively; Table 1). About one third of all participants were female. Participating nephrologists had been specialists for a median of 14 years, while 3 participants did not further specify this question. Years spent practicing as a nephrologist in CKD out-patient care was indicated with a median of 10.5 years and years spent treating out-patients in general with 15.0 years. Participants indicated that they were seeing a median of 30 patients per day of which a median of 11 were CKD pre-dialysis patients. The study population included physicians with both fewer and many years of experience in patient care, indicated by the range of being a specialist (0–35 years) and treating out-patients (0–40 years; Additional file 2: Supplemental Fig. 2), reducing bias through a selection of physicians representing only one level of work experience.

**Table 1:** Characteristics of nephrologists participating in CKDNapp survey (N overall = 54)

**Question**	**N answers (proportion,%)**			
**Age groups (years)**	**51 (100)**			
** ‹40**	6 (11.8)			
** 40–50**	14 (27.5)			
** 51–60**	23 (45.1)			
** ›60**	7 (13.7)			
** ns**	1 (2.0)			
**Sex**	51 (100)			
** male**	33 (64.7)			
** female**	17 (33.3)			
** diverse**	0			
** ns**	1 (2.0)			
		**Values**		
		min–max	mean (sd)	median (p25; p75)
**Working as a specialist in years:**	51 (100)	0–35	14.4 (8.6)	14.0 (8; 22)
**Practicing out-patient CKD care in years:**	43 (100)	0–26	11.4 (8.2)	10.5 (2; 20)
**Treating out-patients in years:**	45 (100)	0–40	15.4 (9.9)	15.0 (10; 21)
**Treating patients / day:**	43 (100)	5–100	36.0 (23.2)	30.0 (15; 50)
**Treating CKD patients / day:**	45 (100)	2–80	22.6 (20.8)	11.0 (8; 40)

*Legend:* *ns* not specified, *sd* standard deviation, *p25* 25^th^ percentile, *p75* 75^th^ percentile

### Module 1: nephrologists’ experiences with CDSS in general

Overall, current CDSS use was poor (81.2% never/rarely, *N* = 39; Table 2). The most frequently indicated reason (Table 2) was missing knowledge about CDSS (43.2%, *N* = 19), followed by no need for systems tied with other reasons (both 20.5%, *N* = 9), and lack of availability of good systems (15.9%, *N* = 7).

**Table 2:** Overview of answers to questions on experiences with CDSS (module M1)

**Questions**	**Answers** N (proportion %)										
				**daily**	**weekly**		**monthly**	**rarely**	**never**		**overall**
**M1.1** how often are you using CDSS in your daily routing				3 (6.3)	5 (10.4)		1 (2.1)	16 (33.3)	23 (47.9)		48 (88.9)
	***not needed***	***no good systems***		***no know-ledge***	***impractical***	***no advent-ages***	***no comprehensible advice***		***other reasons***		
**M1.2** What are reasons for not using CDSS	9 (20.5)	7 (15.9)		19 (43.2)	4 (9.1)	4 (9.1)	1 (2.3)		9 (20.5)		44 (88.9)
		***not applicable***	***mostly not applicable***			***applies partially***	***mostly applicable***			***completely correct***	
**M1.3** I feel confident using CDSS.		18 (38.3)	10 (21.3)			1 (2.1)	11 (23.4)			3 (6.4)	47 (81.5)
**M1.4** I am knowledgable in the way CDSS can be applied in patient care.		16 (34.8)	10 (21.7)			9 (19.6)	8 (17.4)			1 (2.2)	46 (88.9)
**M1.5** I find that a CDSS with personalized information and prognosis about CKD in general is helpful.		1 (2.1)	3 (6.4)			3 (6.4)	18 (38.3)			20 (42.6)	47 (88.9)
**M1.6** A personalized CDSS could be helpful in treating my patients with CKD.		1 (2.1)	2 (4.3)			9 (19.2)	16 (34.0)			17 (36.2	47 (88.9)
**M1.7** A CDSS could improve the quality of patient care.		2 (4.4)	0 (0.0)			10 (21.7)	15 (32.6)			18 (39.1)	46 (88.9)
**M1.8** A CDSS could improve the efficiency of my work.		1 (2.2)	4 (8.7)			10 (21.7)	20 (43.5)			9 (19.6)	46 (88.9)
**M1.9** Using a CDSS would fit into my daily routine.		2 (4.3)	4 (8.5)			15 (31.9)	13 (27.7)			8 (17.0)	47 (88.9)
**M1.10** Would you be willing to try out an available CKD patient CDSS?		1 (2.1)	1 (2.1)			8 (17.4)	14 (30.4)			20 (43.5)	46 (88.9)

*Legend:* All answers are reported as N (%). *CDSS* clinical decision support software, *CKD* chronic kidney disease. M1.1: N_missing_ = 6; M1.2: N_missing_ = 10; M1.3: N_missing_ = 7; M1.4: N_missing_ = 8; M1.5: N_missing_ = 7; M1.6: N_missing_ = 7; M1.7: N_missing_ = 8; M1.8: N_missing_ = 8; M1.9: N_missing_ = 7; M1.10: N_missing_ = 8

Only few participants evaluated CDSS as being impractical (9.1%, *N* = 4), and without advantages for patient care (9.1%, *N* = 4) or giving out incomprehensible advice (2.3%, *N* = 1). More than half of all participants did not feel confident in using CDSS (59.6%, *N* = 28) or were not sure how to apply CDSS in patient care (56.5%, *N* = 26; Table 2). Nevertheless, there was a high confidence that the implementation of a CDSS would be helpful (personalized information/personalized prognosis: 80.9%, *N* = 38; personalized treatment: 70.2%, *N* = 33) and might improve quality of patient care (71.7%, *N* = 33) and work efficiency (63.1%, *N* = 29). The lowest ratings were observed for the question regarding fitting to daily routine (44.7%, *N* = 21). However, most participant were willing to try out an available CDSS (73.9%, *N* = 34).

### Module 2: nephrologists’ expectations towards a helpful CDSS

Next, expectations towards a helpful CDSS were inquired (Additional file 1: Supplemental Table 3; Table 3). The surveyed nephrologists rated as most important CDSS features: the incorporation of guidelines for the treatment of CKD patients (95.6%, *N* = 43; M2.1) as well as the provision of any other important secondary information needed for patient care (97.8%, *N* = 44; M2.2), like specific guidelines or information on rarer diseases underlying the etiology of CKD.

**Table 3:** Overview of answers to questions on expectations of CDSS in general (module M2)

**Questions**	**Answers** N (proportion %)						
**An optimal CDSS should…**	**not applicable**	**mostly not applicable**	**applies partially**		**mostly applicable**	**completely correct**	**overall**
**M2.1** …incorporate all newest guidelines for CKD and it's comorbidities, which should be easily accessible.	0 (0.0)	0 (0.0)	2 (4.4)		7 (15.6)	36 (80.0)	45 (83.3)
**M2.2** …be able to provide fast and easy access to other important secondary information for patient care, if needed.	0 (0.0)	0 (0.0)	1 (2.2)		7 (15.6)	37 (82.2)	45 (83.3)
**M2.3** …contain a clear review of all patient data from any before visits (patient history, lab values, diagnoses, …).	0 (0.0)	6 (13.3)	5 (11.1)		8 (17.8)	26 (57.8)	45 (83.3)
**M2.4** …not change the way I am organizing my workday.	1 (2.2)	4 (8.9)	23 (51.1)		12 (26.7)	5 (11.1)	45 (83.3)
		***cell phone***	***tablet***	***computer***	***other***	***no preference***	
**M2.5** Which device would you preferably use when applying a CDSS?		7 (15.6)	3 (6.7)	30 (66.7)	4 (8.9)	1 (2.2)	45 (83.3)

*Legend:* All answers are reported as N, (proportion in %). *CDSS* clinical decision support software, *CKD* chronic kidney disease. Number of missings for all questions was 9

Participants were undecided about changing their working routine to incorporate a CDSS into their workday (partially applies 51.1%, *N* = 23; M2.4). Details for questions 1 to 4 of module 2 (M2.1-M2.4; see above) with percentages for answer possibilities “*mostly and completely true*” are graphically displayed in Additional file 2: Supplemental Fig. 3. Overall, most participants were in favour of using a desktop computer with internet browser to query a potential CDSS (67%, *N* = 30, Additional file 1: Supplemental Table 3; Additional file 2: Supplemental Fig. 4).

### Module 3: evaluation of the importance/usefulness of prediction algorithms

Table 4 shows numbers and percentages for all answer possibilities concerning possible prediction algorithms incorporated into CDSSs for nephrologists. Here, participants were mostly interested in the prediction of CKD progression and prediction of an AKI event. Prediction of stroke, a peripheral arterial occlusive disease (PAOD) event, and mortality were rated as being less important, but still of high interest (Additional file 1: Supplemental Table 4).

**Table 4:** Overview of answers concerning the evaluation of potential features of interest within a CDSS in particular (module M3)

**Questions**	**Answers** N (proportion %)					
Rate the following prediction algorithms	**un-important**	**mostly not important**	**neutral**	**mostly important**	**very important**	**overall**
**M3.1** AKI event	0 (0.0)	0 (0.0)	2 (4.4)	15 (33.3)	28 (62.2)	45 (83.3)
**M3.2** CKD progression	0 (0.0)	0 (0.0)	1 (2.2)	9 (20.0)	35 (77.8)	45 (83.3)
**M3.3** stroke event	0 (0.0)	1 (2.3)	8 (18.2)	19 (43.2)	16 (36.4)	44 (81.5)
**M3.4** PAOD event	0 (0.0)	3 (6.7)	6 (13.3)	23 (51.1)	13 (28.9)	45 (83.3)
**M3.5** mortality	0 (0.0)	2 (4.6)	6 (13.6)	21 (47.7)	14 (31.8)	44 (81.5)
**M3.6** access to guidelines	0 (0.0)	1 (2.2)	2 (4.4)	8 (17.8)	34 (75.6)	45 (83.3)
**M3.7** in-silico mode	0 (0.0)	0 (0.0)	1 (2.3)	15 (34.1)	28 (63.6)	44 (81.5)
			**yes**	**no**	**maybe**	
**M3.8** Would you be willing to test a first version of a CDSS?			38 (84.4)	0 (0.0)	7 (15.6)	45 (83.3)

*Legend:* All answers are reported as N, (proportion in %). *CKD* chronic kidney disease, *PAOD* peripheral arterial occlusive disease, *CDSS* clinical decision support software

The access to guidelines was rated as being highly important (93.3%, *N* = 42) as well as an in-silico modification for virtually changing patient features in order to explore their influence on potential future outcomes (97.7%, *N* = 43; Table 4). Most participant were in favour of testing a CDSS incorporating the evaluated features (84.4%, *N* = 38; Table 4).

### Module 4: ethical aspects with regard to CDSS

Answers to seven questions on ethical aspects of CDSS were mostly diverse (Additional file 1: Supplemental Table 5). Participants agreed that CDSS would not reduce trust of patients into their treating physicians (80.0%, *N* = 24) and most participants also did not believe that application of CDSS would lead to discrimination of some patient groups (68.9%, *N* = 24). Statements being assessed as partially or mostly true were that certain parts of a physician’s work will be taken over by CDSS in the future (66.7%, *N* = 21) and that CDSS are more helpful for inexperienced doctors (57.8%, *N* = 13), but about 50% of participants did not share this opinion, respectively. About 67% of all participants did not believe that introduction of CDSS will lead to a reduction in a physicians clinical abilities. Mixed answers were given for question 4 of this module with one third of participants believing that CDSS will lead to a shift for doctors towards a more patient-centered medicine in the future, one third of participants partially agreeing and one third of participants not agreeing. Similarly mixed answers were given to the question whether missing transparency of advice given by CDSS is perceived as a problem. Here, about 30% of participants stated that this was mostly not true, but about 44% of participants believed this to be partially or mostly true.

## Discussion

Our survey evaluated answers given by nephrologists from Germany regarding their awareness of existing CDSS, their openness to use CDSS, their knowledge of how CDSS can assist them in everyday patient care, and an assessment of prediction algorithms as well as literature support being part of a future nephrologists’ CDSS. The work-up of this survey will help in addressing possible barriers for implementation of CDSS in a nephrologist’s medical routine and will therefore facilitate an optimal development of CDSS in the nephrology field. Past studies have shown that the identification of a specific target group is an important aspect of CDSS design. Notably, specialty physicians reported an increased need for a knowledge source that helps to answer complex context specific clinical questions, which implies that they are more likely to benefit from a specified CDSS [34]. Surveys are a useful and valuable tool in determining needs of researchers developing tools, CDSSs on the one hand and research users/physicians on the other hand [35].

In everyday clinical practice, treating nephrologists are confronted with a large amount of patient data as well as a multitude of research papers and guidelines. Moreover, patient care needs to be individualized as well as based on these newest guidelines and extensive research has been done on the facilitators and barriers of adherence to guidelines in patient care [36, 37]. In recent years, a growing number of mobile-health technology in the management of chronic diseases has been observed [38, 39]. These applications are mainly aimed at patients as end-users and primarily achieve pure data collection [40]. The integration of data accumulated through these apps in clinical nephrology practices is limited by: 1. A diversity of programs, 2. Data entries, that are optimized to the patient perspective and not the physician’s perspective, 3. A lack of compatibility with clinical data/data processing software (adequate infrastructure) [41]. In addition, highly sought after and need of prediction algorithms of possible adverse events and disease progression require models developed and validated in CKD patient cohorts. Moreover, a transparent presentation of outcomes from these prediction models is of high importance for the treating nephrologist. Taken together, these limitations have led to low ratings of CDSS use by nephrologists [42, 43]. Early integration of physicians into the development of a CDSS is therefore essential for later implementation in clinical practice.

### Nephrologists’ experiences with CDSS in general

Little is known about the experiences of German nephrologists with CDSS. Our survey showed that 80% of all participants rarely or never use CDSSs, but about 80% of participants would find a personalized CDSS helpful for CKD patient care and over 70% of participants believe that it would improve patient care as well as their work efficiency. In the past, many examples of helpful CDSSs can be found supporting this notion [16, 44–47]. Taken together with the knowledge that specialty physicians in particular profit from CDSS support [34], the development of a specialized CDSS for nephrologists supporting them in personalized CKD patient care, seems overdue. In our survey we focus on first general aspects of CDSS to then focus on special aspects of possible CDSS in the form of prediction algorithms. Medication management, diagnostic tools, and classification systems can also be a part of CDSS. However, we did not focus on in these aspects in our survey.

### Nephrologists’ expectations towards a helpful CDSS

In our survey, most nephrologists generally agreed that the most important topics are accessibility to newest guidelines and other important secondary information essential for CKD patient care, but also management of patient data from previous visits. This is in line with data obtained from other groups working on facilitators for the implementation of CDSSs. Varonen et al. were able to show that physicians are mostly interested in managing the mass and complexity of information available when thinking of CDSSs [48]. It will be important to differentiate the expectations of nephrologists on how guidelines will be implemented into a CDSS. Having access to an uploaded PDF guideline might be less comfortable since one will have to go through the guideline and decide what will fit the patient in question best; alternatively, implementation of guideline recommendations would go along with easier accessibility, but also with restriction to the given recommendation suggested by the CDSS. Another important aspect of this module was the resistance to change of a nephrologist’s workday. Here, 40% of all participants were opposed to any changes, an aspect that has been seen during the implementation process of CDSS before [49], bringing up the necessity to carefully describe and collect contextual factors important for practice changes before CDSS implementation [50, 51]. Overall, nephrologists did recognize the usefulness of CDSS, which is a common theme on the perception of CDSS by health care professionals [52]. But long-term implementation of CDSS may pose a challenge that might be tackled by the implementation of behavior change interventions [53].

### Evaluation of the importance/usefulness of prediction algorithms within CDSS

CKD patients constitute a heterogeneous group, making the development of useful prediction models challenging. The development of a clinical prediction model requires a collaborating team of experts in statistics, well qualified in the specific methodology, computer science, researchers and physicians to identify key elements of the model [54]. Predicting outcomes in everyday practice can help guide clinical care, decision making, and resource allocation. In fact, prediction of CKD progression and AKI were rated the highest by participating physicians. Several prediction models have been developed for CKD before, however, are currently infrequently used [55]. Reasons could be that few of them have been appropriately externally validated and out of 23 models only 2 met the proposed criteria [56, 57] for clinical usefulness [55]. The best-known model, the Kidney Failure Risk Equation by Tangri et al. [16], uses 8 routine laboratory parameters. Zacharias et al. [15] established a new risk equation based on data from the GCKD study using six routinely available laboratory parameters and validated the algorithm in 3 independent prospective observational cohort studies. There are tools for useful clinical prediction models to guide development and ensure transparent reporting (TRIPOD [58]: Transparent Reporting of a Multivariable Prediction Model for Individual Prognosis or Diagnosis).

Acute kidney injury (AKI) is associated with short- and long-term mortality in hospitalized patients (particularly, if patients are admitted to the intensive care unit [59]) and increased risk of major adverse kidney events including CKD progression [10, 60, 61]. Current international guidelines recommend follow-up of all patients with AKI for 90 days [62]. Recently, a study from Sawhney et al. indicated benefits for re-empting readmissions, death, and subsequent CKD progression for follow-up after AKI [63]. Risk-stratifying CKD patients for AKI has potential to facilitate targeted interventions, thus, physicians were second most interested in an AKI prediction model. Overall, data from large CKD observational studies on the relationship between AKI and CKD are scarce. Identifying patients for increased risk can improve disease management.

The risk of CVD events is higher in patients with CKD than in the general population. However, few models exist to predict the risk of CVD events in patients with CKD to date. Although prediction of CVD in general was not queried in this survey, but stroke and PAOD as secondary complications of CVD were included (M3.3, M3.4). In addition to disease etiology and comorbidities, modifiable lifestyle factors of CKD play an important role in primary prevention [64, 65]. Current CKD management guidelines recommend that patients adhere to a healthy lifestyle including diet with low potassium and low salt intake, physical activity, abstain from tobacco use, and limited alcohol consumption [66, 67]. Adherence to healthy dietary patterns is associated with lower risk for CKD progression and all-cause mortality in CKD [68, 69], however, long-term behaviour change of patients remains a challenge in practice. CKD is a silent disease, i.e., it causes few symptoms, which negatively affects the motivation of patients to change their behaviour. Successfully addressing this problem involves ensuring adequate time spent on conceptualization and planning, as well as visualization of goals with regular adjustment involving the patients.

### Ethical aspects with regard to CDSS

CDSS and usage of machine learning techniques will lead to profound changes within healthcare. This also gives rise to many ethical questions, since high-quality patient treatment does not only depend on the correct prediction, prevention or improved decision making by CDSS containing a better selection of individual treatments, but also on experience, empathy, and the concrete judgement of physicians [70]. In this survey, nephrologists were not worried about a reduction in trust from patients when applying and using CDSS. This might be due to the fact, that both patients and physicians share the view that the final act of decision making rests with the human actors. CDSS cannot carry out the full process of clinical care, making CDSS supportive systems to be used by physicians not vice versa also in the future [71]. Moreover, advice by CDSS does not necessarily have to be followed but there is a complex interaction between expert system advice and physicians’ own judgment and discretion which affects physicians’ epistemic authority, particularly in situations of “peer disagreement” [72]. Nonetheless, past research endeavors were able to show that the adoption of CDSS based on clinical practice guidelines (= systematically developed statements to assist practitioners and patient decisions about appropriate health care for specific clinical circumstances) promoted adherence to these guidelines and led to improved patient management [73]. In this survey, most nephrologists did not believe in a reduction in clinical abilities by applying CDSS. Whether CDSS will open up more time for physicians to actually talk to their patients by freeing them from time-consuming decision making seems to be less clear and nephrologists were ambivalent in their answers. Indeed past studies were able to show, that rigidity of CDSS can lead to time-consuming data entry processes that physicians will come to resent leading to rejection of the system [74] and potentially affecting the patient-physician-relationship and the quality of clinical care in a negative way. Moreover, manual data entry can lead to erroneous data entries resulting in inaccurate CDSS responses [75]. An optimal CDSS for nephrologists would therefore entail an internal data entry check, which would help in recognizing false data entries and give feedback to the physician entering the data into the application. Other challenges that need to be faced are, amongst others, poor data sets and poorly optimized algorithms leading to possible discrimination of population fractions or erroneous decision making for underrepresented patient groups. In our survey, only about 11% of participants were worried that this might be a problem of CDSS. A study by Mitchell et al., by contrast, could show that using CDSS in high-minority hospitals had higher quality improvements when focusing on CDSS adoption [76]. Nonetheless, underrepresentation of minority groups in clinical trials is a well-known problem [77] and may lead to maladjusted algorithms for these groups concerning CDSS-based prediction of adverse events.

### Strengths and Limitations

This study has important strengths and limitations. Since we did not have a tracking system to indicate respondents from non-respondents, we could not compare characteristics of those not included in the study. Having two methods to receive answers from participants, namely telephone interview or do-it-yourself, might also have introduced bias as participants might have been less likely to not give answers for some of the questions. We were only able to acquire a small number of nephrologists compared to the number of nephrologists practicing in the outpatient setting in Germany (2021: *N* = 1125) willing to participate in our study, which potentially introduced bias. Nevertheless, our study population reflected demographics of nephrologists in Germany well with higher number of males mostly in an age range between 51–60 years (https://www.bundesaerztekammer.de/baek/ueber-uns/aerztestatistik/2022; https://www.gbe-bund.de/gbe/pkg_olap_tables.prc_set_hierlevel?p_uid=gasta&p_aid=21479372&p_sprache=D&p_help=2&p_indnr=96&p_ansnr=47431095&p_version=3&p_dim=D.489&p_dw=44485&p_direction=drill.). Our study, however, has multiple strengths. This is the first survey of nephrologists exploring their CDSS knowledge and requirements to contemporary needs across Germany. We examined readiness in terms of infrastructure availability and interest to test the instruments for use in their own practice.

## Conclusion

This survey provides insights into experience with and expectations of outpatient nephrologists on CDSS in general and prediction algorithms, literature support, and in-silico-modification possibilities of CDSS in particular. Despite the current lack of knowledge on CDSS, the willingness to integrate CDSS into daily patient care, and trying out specialized CDSS in the future was high. This is underlined by the readiness to test a CDSS prototype focused on CKD patient care.

### Supplementary Information

**Additional file 1: Supplemental Table 1.** CKDNapp survey questionnaire questions and answer possibilities. **Supplemental Table 2. **Complete numbers and percentages to questions on experiences with clinical decision support software of module 1. **Supplemental Table 3.** Complete numbers and percentages to questions on expectations of a clinical decision support software in general of module 2. **Supplemental Table 4.** Complete numbers and percentages to questions on prediction algorithms of module 3. **Supplemental Table 5.** Complete overview of questions on ethical aspects of CDSS from module 4.

**Additional file 2: Supplemental Fig. 1.** CKDNapp survey analysis set and number of missingness per participant across all questions. **Supplemental Fig. 2.** Scatterplots of participants concerning their work experience. **Supplemental Fig. 3.** Expectations of a clinical decision support software in general of module 2 – details on questions M2.1 to M2.4. **Supplemental Fig. 4.** Preferred device for querying a CDSS.

## Data Availability

Public posting of individual level participant data is not covered by the informed patient consent form. All summary statistics necessary to interpret, replicate, and build upon the results reported in the article are provided in anonymized form in the publication.

## Abbreviations

AKI: Acute kidney injury
BMBF: German Federal Ministry of Education and Research
CDSS: Clinical decision support systems
CKD: Chronic kidney disease
CKDNapp: CKD Nephrologist App
DESIREE: Decision Support In Routine and Emergency Health
eGFR: Estimated glomerular filtration rate
GCKD: German Chronic Kidney Disease study
M: Module question
ns: Not specified
p25: 25^Th^ percentile
p75: 75^Th^ percentile
REDCap: Electronic Data Capture System
sd: Standard deviation
SP: Subproject
TAM2: Technology Acceptance Model
