# Supplementary material for: Expectation of clinical decision support systems: a survey study among nephrologist end-users
Source: BMC Med Inform Decis Mak. 2023 Oct 26;23:239. doi: 10.1186/s12911-023-02317-x (PMC10605935; doi:10.1186/s12911-023-02317-x)

**Expectation of clinical decision support systems: Survey study among nephrologist end-users**

**SUPPLEMENTAL FIGURES**

Fruzsina Kotsis^1,2*^, Helena Bächle^1*^, Michael Altenbuchinger^3^, Jürgen Dönitz^3,4^, Yacoub Abelard Njipouombe Nsangou^4^, Heike Meiselbach^5^, Robin Kosch^3^, Sabine Salloch^6^, Tanja Bratan^7^, Helena U. Zacharias^8^, Ulla T. Schultheiss^1,2^

^1^Institute of Genetic Epidemiology, Faculty of Medicine and Medical Center - University of Freiburg, Freiburg, Germany

^2^Department of Medicine IV – Nephrology and Primary Care, Faculty of Medicine and Medical Center - University of Freiburg, Germany

^3^Department of Medical Bioinformatics, University Medical Center Göttingen, Göttingen, Germany

^4^Institute of Computational Biology, Helmholtz Zentrum München, Germany ^5^Department of Nephrology and Hypertension, University Hospital Erlangen, Friedrich-Alexander-Universität Erlangen-Nürnberg, Erlangen, Germany

^6^Institute for Ethics, History and Philosophy of Medicine, Hanover Medical School, Hanover, Germany

^7^<https://www.isi.fraunhofer.de/>, Karlsruhe, Germany

^8^Peter L. Reichertz Institute for Medical Informatics of TU Braunschweig and Hannover Medical School, Hannover, Germany.

*the first authors contributed equally to this project

**Table of Contents**

SupplementaL Figures 3-6

Supplemental Figure 1: CKDNapp survey analysis set and number of missingness per participant across all Questions 3

Supplemental Figure 2: Scatterplots of participants concerning their work experience 4

Supplemental Figure 3 Expectations of a clinical decision support software in general of module 2 – details on questions M2.1 to M2.4. 5

Supplemental Figure 4: Preferred device for querying a CDSS. 6

**Supplemental Figure 1**: CKDNapp survey analysis set and number of missingness per participant across all questions.

**B**

**A**


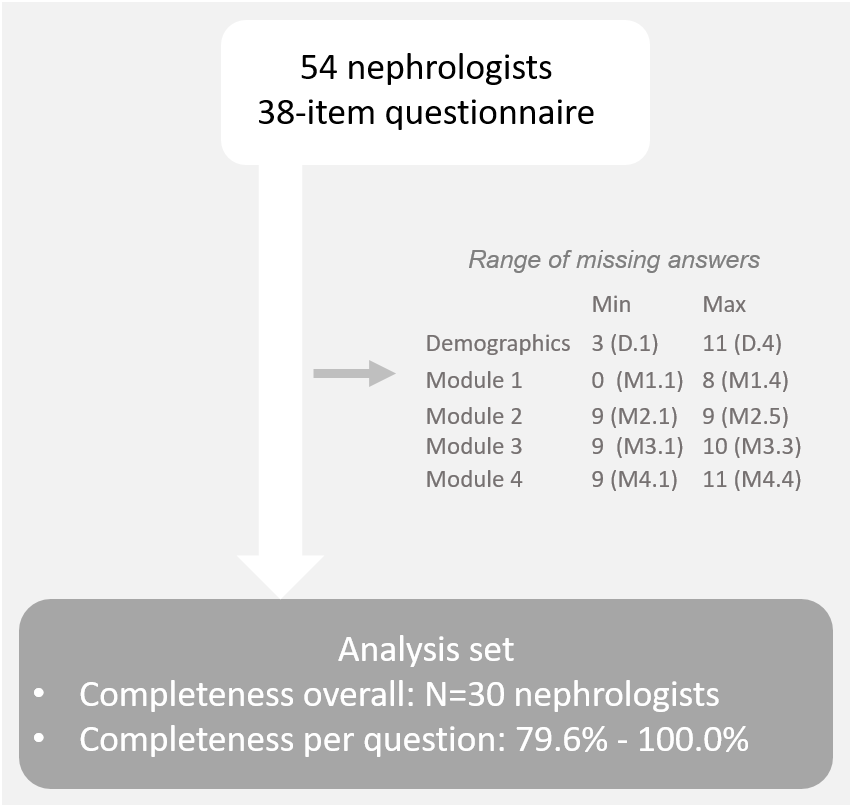

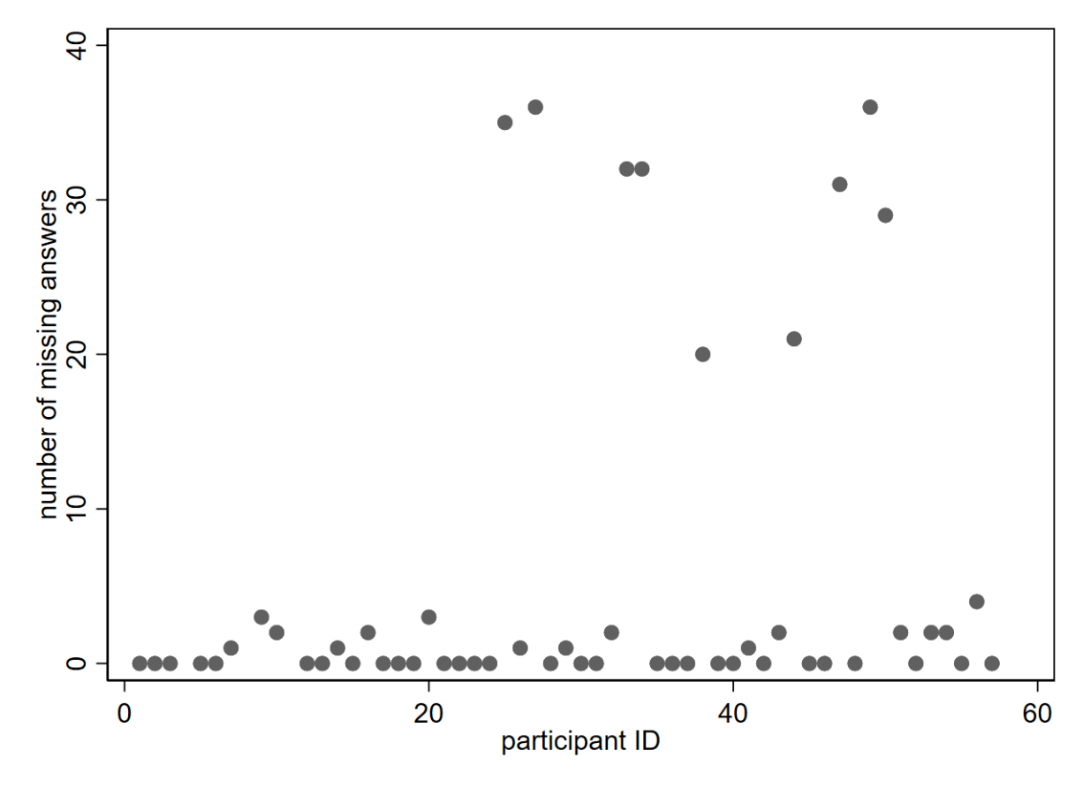


**Legend:**

**A:** analysis set with number of missingness per module. **B:** Scatterplot of number of missingness values per participant across all questions.

**Supplemental Figure 2**: Scatterplots of participants concerning their work experience

A: years spent treating out-patients

B: years being a consultant


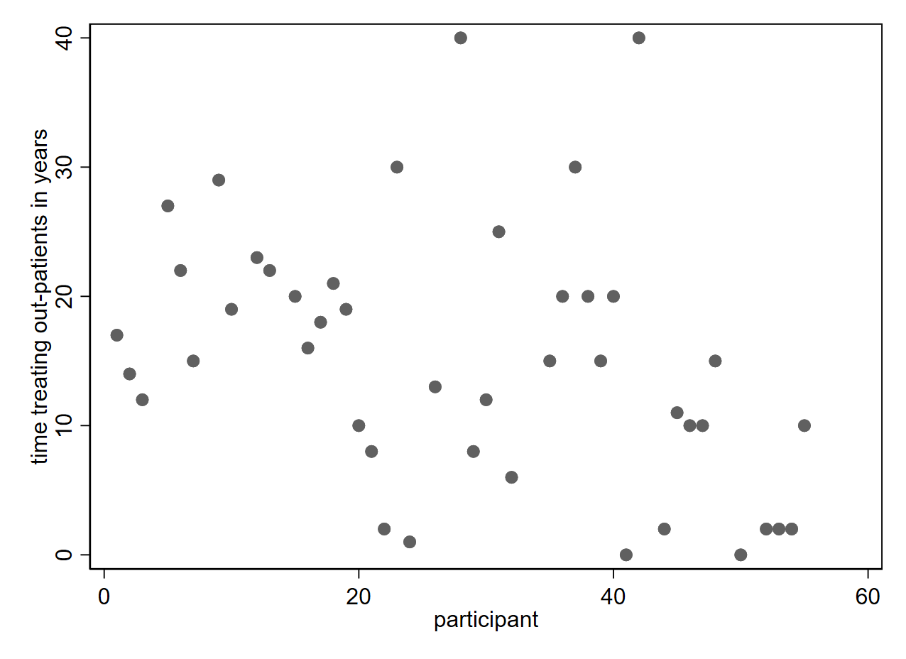

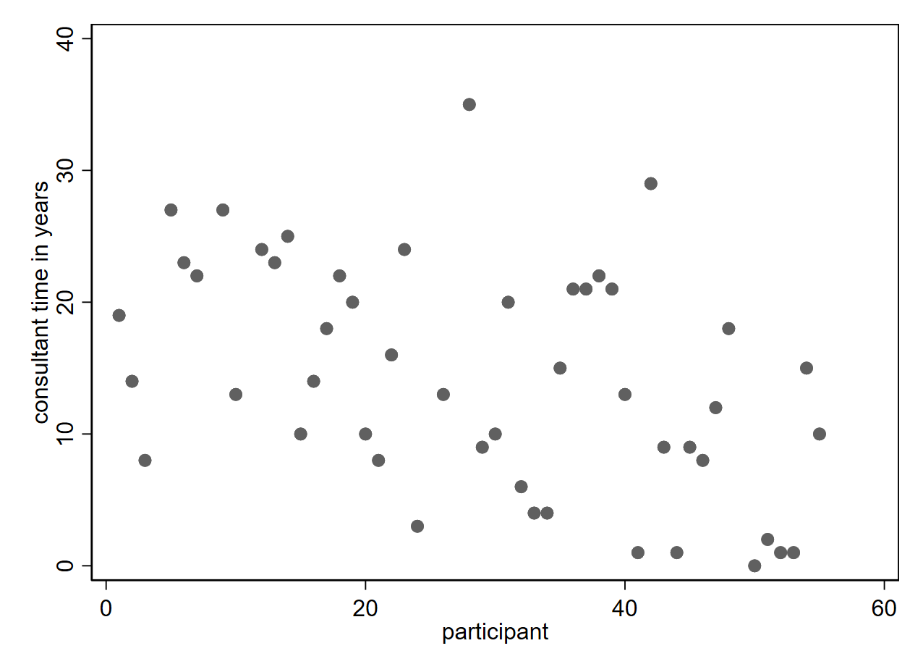

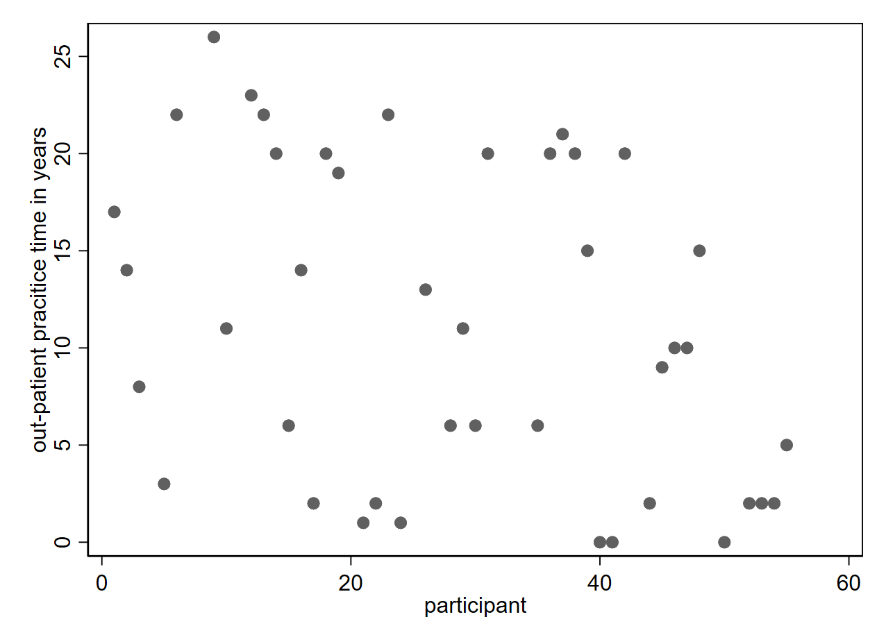
**Supplemental Figure 3**: Expectations of a clinical decision support software in general of module 2 – details on questions M2.1 to M2.4.

C: years spent in an out-patient practice


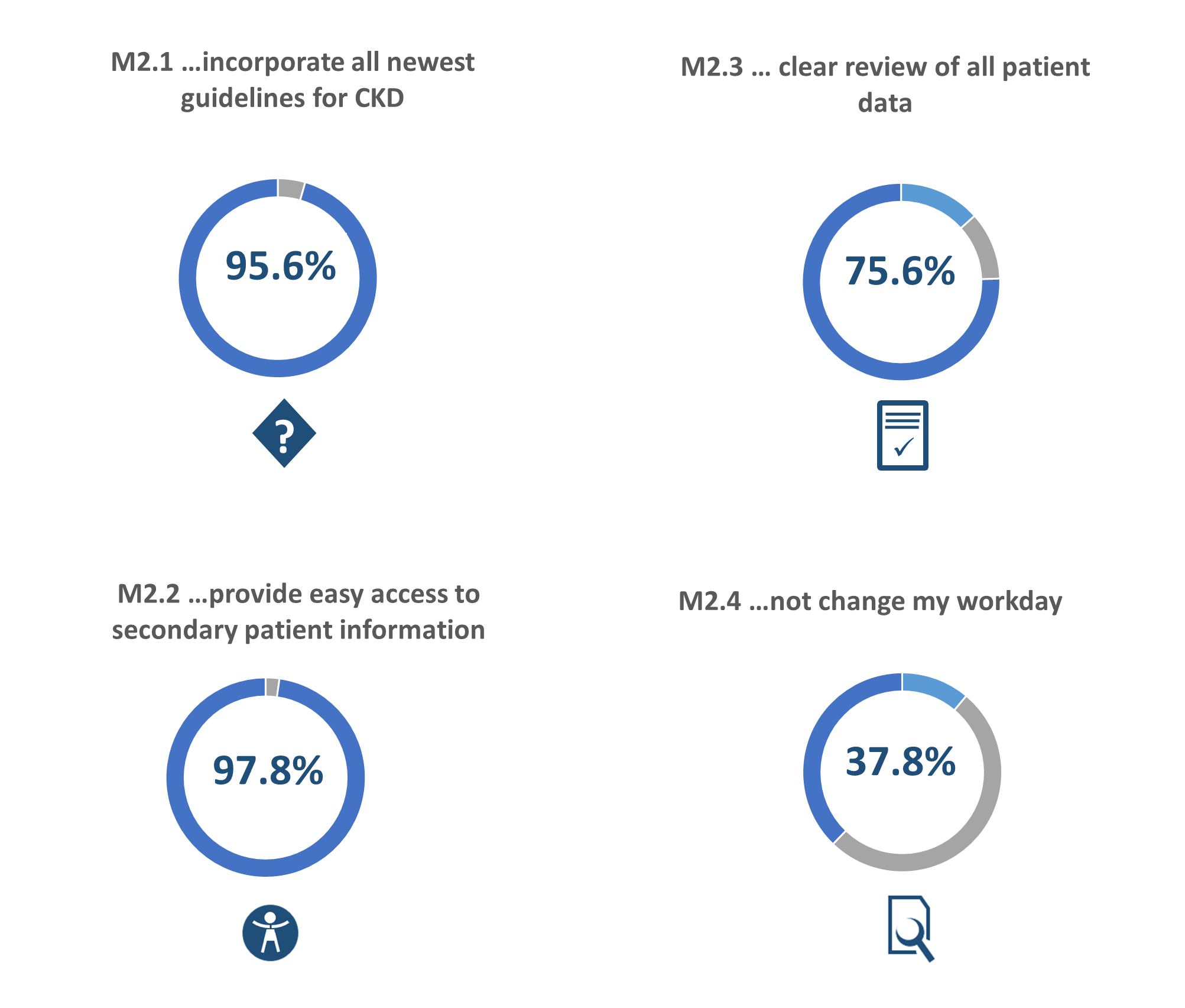


**An optimal CDSS should…**


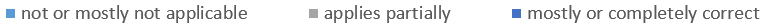


Legend: percentages refer to the summary of percentages given for answer possibilities: mostly and completely correct. For M2.1 to M2.4: N_missing_=9, N_notspecified_=0, N_answers_=45. Percentages for all answers can be found in **Supplemental Table 3, Additional file 1**.

**Supplemental Figure 4**: Preferred device for querying a CDSS.


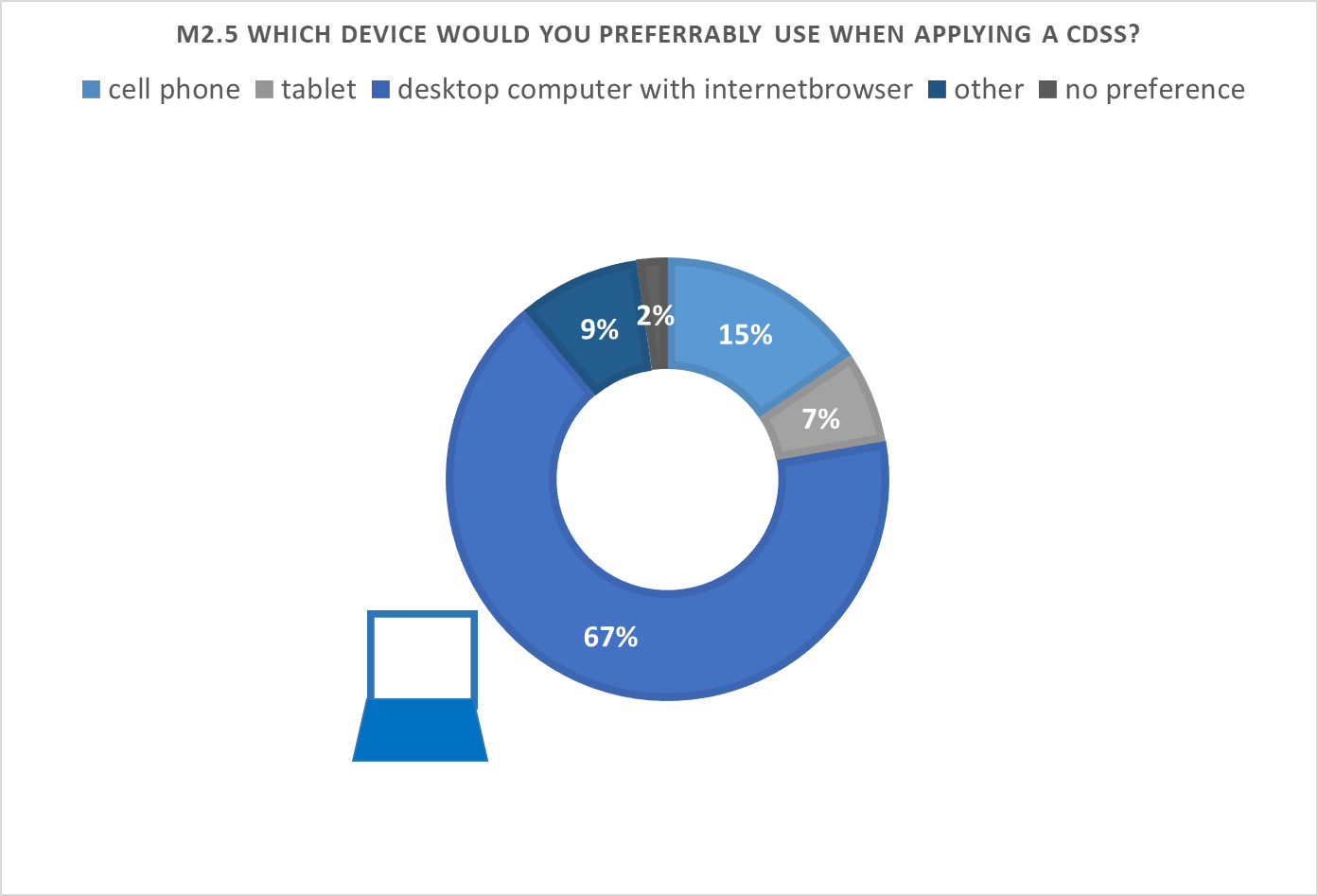

Supplement: Supplementary file 2 — Additional file 2: Supplemental Fig. 1. CKDNapp survey analysis set and number of missingness per participant across all questions. Supplemental Fig. 2. Scatterplots of participants concerning their work experience. Supplemental Fig. 3. Expectations of a clinical decision support software in general of module 2 – details on questions M2.1 to M2.4. Supplemental Fig. 4. Preferred device for querying a CDSS. [file 12911_2023_2317_MOESM2_ESM.docx]
